# Paraganglioma and cardiomyopathy leading to cardiogenic shock: a case report

**DOI:** 10.1093/eschf/xvag032

**Published:** 2026-01-21

**Authors:** Esther Y Son, Mariam M Ardehali, Benjamin M Moy, Chengwei Peng, Anjan Tibrewala

**Affiliations:** Northwestern University Feinberg School of Medicine, Chicago, IL, USA; Department of Medicine, Northwestern University Feinberg School of Medicine, Chicago, IL, USA; Department of Medicine, Northwestern University Feinberg School of Medicine, Chicago, IL, USA; Division of Hematology/Oncology, Department of Medicine, Northwestern University Feinberg School of Medicine, Chicago, IL, USA; Division of Cardiology, Department of Medicine, Northwestern University Feinberg School of Medicine, 676 N St Clair St, Suite 600, Chicago, IL 60611, USA

**Keywords:** Cardiogenic shock, Heart failure, Pheochromocytoma, Paragangliomas

## Introduction

The annual incidence of pheochromocytomas and paragangliomas (PGGLs) in the USA is between 500 and 1600 cases.^1^ Pheochromocytomas are neoplasms of the chromaffin cells of the adrenal medulla, whereas extra-adrenal pheochromocytomas are called PGGLs, referred together as pheochromocytomas and PGGLs.^2,3^ Pheochromocytomas and PGGLs both release catecholamines (i.e. epinephrine and norepinephrine) and can result in hypertension, arrhythmias, and structural changes, which may cause non-ischaemic cardiomyopathy (NICM), takotsubo syndrome (TS), hypertensive heart disease, and myocarditis.^3,4^ Although tumour resection can lead to improvements, chronic catecholamine exposure may lead to irreversible myocardial damage and heart failure with reduced ejection fraction (HFrEF).^5^

In certain cases, stage D (i.e. advanced) heart failure (HF) therapies including heart transplant or durable left ventricular assist device (LVAD) implantation may be considered.^6^ However, eligibility for these therapies in patients with PGGLs has unique considerations. Furthermore, management strategies for cardiogenic shock in patients with PGGLs are complicated and not clearly reported in the current literature. This case study (i) describes the clinical dilemma of a patient whose advanced HF precluded surgical resection due to cardiovascular risk profile while the presence of a PGGL precluded eligibility for advanced HF therapies and (ii) illustrates medical management strategies for cardiogenic shock in the setting of a known PGGL and Stage D HFrEF.

## Case

**Figure 1:**
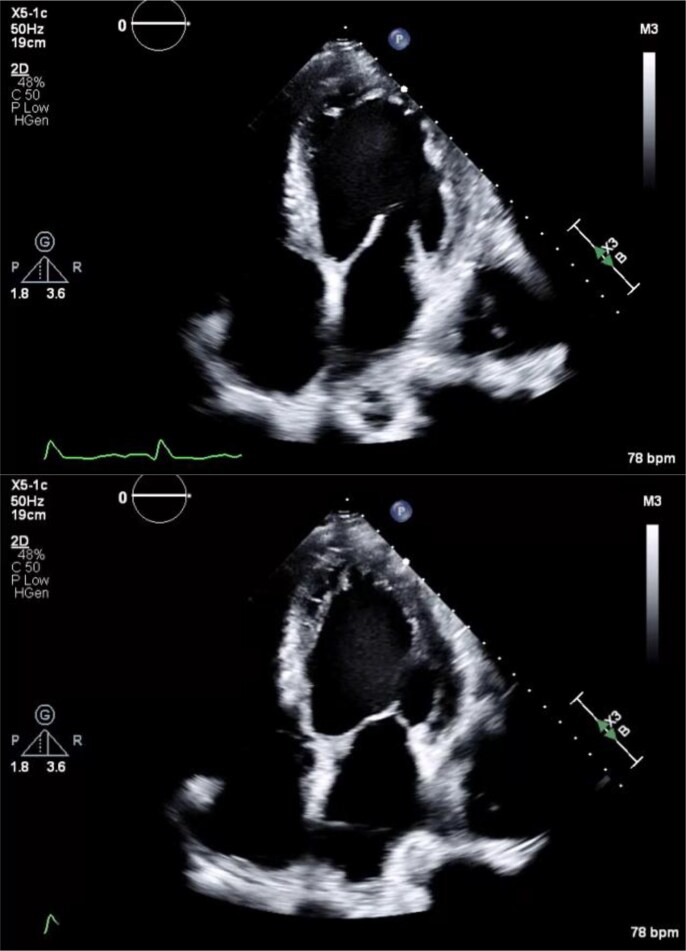
Transthoracic echocardiogram demonstrating severely dilated and dyskinetic left ventricle during diastole (top) and systole (bottom) for comparison

A 66-year-old man with a past medical history significant for Stage D HFrEF (EF 15%) on home milrinone 0.25 μg/kg/min, chronic kidney disease, coronary artery disease (CAD) complicated by non-ST-elevation myocardial infarction (NSTEMI) with bare metal stenting (BMS) in 2010, ST-elevation myocardial infarction with BMS in 2013, NSTEMI in 2015, paroxysmal atrial fibrillation, and para-aortic PGGL presented in cardiogenic shock. He was febrile to 38.2°C with a regular heart rate and hypertensive to 215/118. He was in respiratory distress requiring bilevel positive airway pressure (BiPAP) for increased work of breathing and hypoxaemia. Initial laboratory findings were significant for troponin 175 pg/mL, white blood cell count 15 × 10^3^/μL with a left shift, lactic acid 4 mmol/L, creatinine 3.5 mg/dL (baseline 2.4 mg/dL), and brain natriuretic peptide of 2500 pg/mL. A chest x-ray showed pulmonary oedema, and an electrocardiogram showed a baseline right bundle branch block alternating with a new left bundle branch block. Telemetry showed sinus rhythm with intermittent runs of non-sustained ventricular tachycardia. He was transferred to the cardiac intensive care unit for management of cardiogenic shock. An echocardiogram showed a severely dilated and dyskinetic left ventricle with an EF of 12% and mild to moderate mitral regurgitation (*Figure 1*). Right ventricular size and systolic function were normal with a dilated right atrial cavity and estimated right atrial pressure of 15 mmHg. The patient was given intravenous diuresis for preload optimization. He was maintained on his home milrinone and started on a nitroprusside drip that was weaned within 2 days by starting and titrating hydralazine, isosorbide dinitrate, and doxazosin. With this management, his blood pressure improved to (120–130)/80. An infectious workup was negative, and his fever and leucocytosis resolved.

Of note, the patient had 10 hospitalizations for decompensated HF between 2018 and 2025, many of which included exacerbations likely related to hypertensive urgency. Three months prior to the current admission, cardiac magnetic resonance imaging showed a near transmural infarct in the territory of the right coronary artery, severely dilated left ventricle with EF 16%, moderately dilated right ventricle and reduced systolic function, and moderate mitral regurgitation. After a multi-disciplinary discussion about possible advanced therapies, the patient was determined to be at prohibitive risk for heart transplant or LVAD due to the para-aortic PGGL.

**Figure 2:**
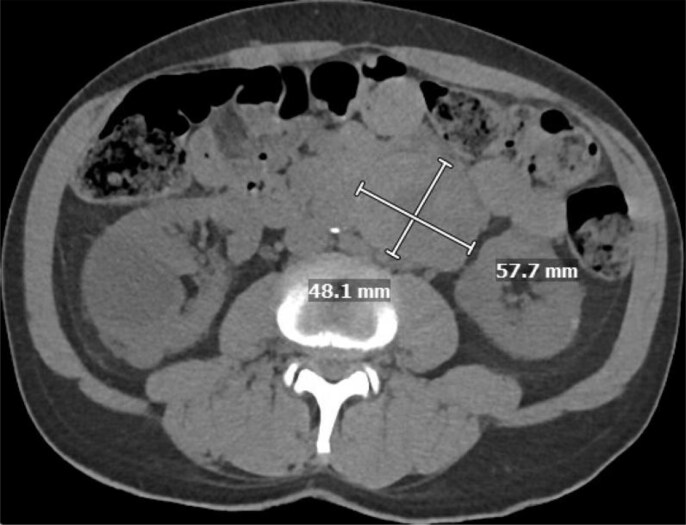
Computed tomography abdomen without contrast demonstrating para-aortic paraganglioma

Regarding his oncologic history, a para-aortic mass was revealed during a hospitalization 7 years prior to this hospitalization for acute decompensated HF (*Figure 2*). His urine normetanephrine at that time measured 1339 pg/mL (normal ≤57 pg/mL) and urine metanephrine measured 1586 pg/mL (normal ≤148 pg/mL). The mass demonstrated positive uptake on iodine meta-iodobenzylguanidine scan, and a positron emission tomography (PET) Dotatate scan was consistent with a somatostatin receptor–positive tumour, most compatible with PGGL. Several months prior to this admission, endocrine surgery was consulted for possible resection of the mass, but the patient was determined to be at prohibitive risk of peri-operative cardiac complications given Stage D HFrEF requiring home inotropes, so he started receiving lanreotide injections. The patient was also prescribed doxazosin, though had been intermittently adherent with the medication.

During the current hospitalization, the patient was diagnosed with a new Type A aortic dissection on imaging, and the surgical risk was deemed prohibitive. The patient was discharged to hospice care.

## Discussion

This patient likely presented with an underlying mixed cardiomyopathy, including extensive ischaemic heart disease and NICM from his PGGL. Most common cardiac manifestations of PGGL cardiomyopathy are non-ischaemic from chronic hypertension or catecholamine-induced myocardial injury.^3,4^ These phenomena are thought to develop in response to catecholamine surges, which lead to hyperactivation of the sympathetic nervous system, cardiac sympathetic nerve terminal disruption, and subsequent myocardial damage.^3^ Ischaemic cardiomyopathy may arise from PGGL-related pathology as well. Recent studies demonstrate that catecholamine phenotype may determine underlying pathophysiological mechanisms of cardiomyopathy in pheochromocytoma and PGGL.^7^ Paragangliomas predominantly secrete norepinephrine and are associated with a higher risk of atherosclerotic cardiovascular complications. In contrast, PGGLs that secrete predominantly epinephrine more often result in paroxysmal hypertension, more frequent tachyarrhythmias, and acute myocardial injury, including Type 2 myocardial infarction and TS rather than atherosclerotic events.^7^ In our patient whose normetanephrines were predominantly elevated, a component of his ischaemic heart disease may be associated with his PGGL.

Pheochromocytoma and PGGL crisis, which is a rare but highly fatal complication of PGGLs, can involve multiple organs and lead to multi-organ failure. Of the complications of PGGL crisis, pheochromocytoma-induced cardiogenic shock is a rare but described phenomenon.^8^ In our patient, cardiogenic shock was likely driven by hypertensive emergency in the setting of his active PGGL.

**Figure 3:**
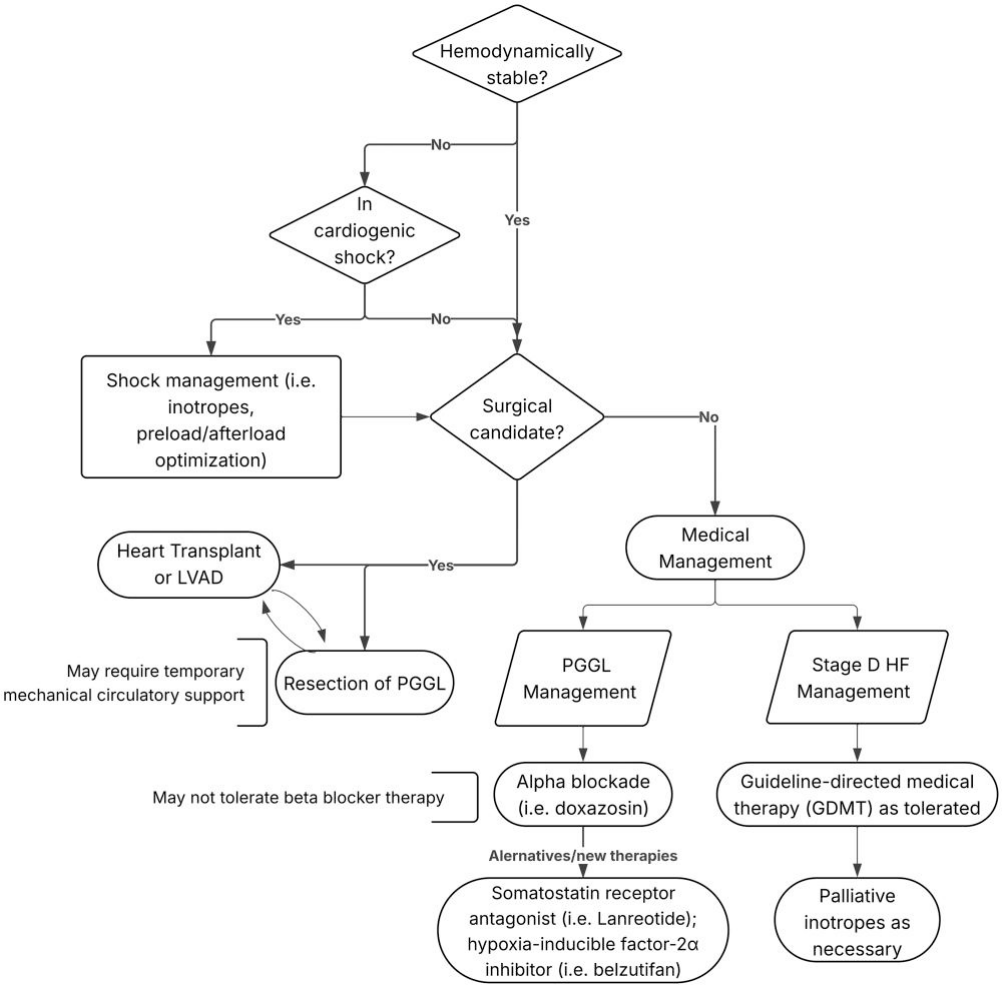
Treatment algorithm for Stage D heart failure with reduced ejection fraction in the setting of pheochromocytoma and paraganglioma

There is paucity of data regarding treatment for cardiogenic shock in setting of PGGL and Stage D HFrEF. Thus, a personalized approach is necessary (*Figure 3*). In this case, we prioritized stabilizing the patient from his PGGL-induced hypertensive emergency with nitroprusside per guidelines for catecholamine-induced hypertensive crises in HF.^9^ Concurrently, we initiated hydralazine and isosorbide dinitrate for afterload and preload reduction given the patient’s HFrEF.^6^ After the patient was more haemodynamically stable, alpha-blockade with doxazosin was initiated to target persistent hypertension due to his PGGL. Of note, this patient could not be initiated on beta-blocker therapy given concomitant use of inotrope therapy. Although alpha-blockers have historically had a relative contraindication in HFrEF due to concern for worsening HF, more recent data may suggest these drugs can safely be used in HFrEF although neurohormonal advantages are lacking.^6^ This case highlights how clinicians should account for all comorbidities in the medical management of HFrEF patients including utilization of medical therapies that fall outside of the four pillars of guideline-directed medical therapy.

Moreover, the team faced a clinical dilemma of managing Stage D HFrEF and PGGL, as the ability of the patient to get definitive therapy for these conditions is limited by the other. Specifically, a PGGL precludes advanced HF therapies, while Stage D HF significantly increases the surgical risk of tumour resection. Unfortunately, the patient likely missed a key window during which tumour resection presented with fewer surgical risks (i.e. first discovery of PGGL in the setting of hypertensive emergencies prior to the progression to advanced HF). Most reports regarding HF and recovery of heart function after pheochromocytoma resection are in younger patients without ischaemic heart disease. Therefore, the likelihood of myocardial recovery for this patient is unclear even if surgical resection was pursued.^4,10–12^

Advances in medical therapy with pre-operative alpha- and beta-blockade, as well as post-operative surveillance strategies, have reduced intra-operative and post-operative complications, but surgical resection remains the primary curative treatment for resectable tumours.^13^ It is also important to consider the location of the PGGL, as mediastinal and cardiac PPGLs that arise from para-aortic or para-vertebral sympathetic ganglia are often more complex and invasive; these may require cardiopulmonary bypass during surgery and have lower complete resection rates than its abdominal and adrenal counterparts.^13^

In a patient who cannot undergo surgical resection of PGGL, newer medical therapies such belzutifan, a hypoxia-inducible factor-2α inhibitor, may be considered. However, no current clinical trials support its use in Stage D HF on inotropes.^14^ Lanreotide, a long-acting somatostatin receptor antagonist that this patient had been receiving, has been shown in a recently presented clinical trial to have a favourable safety profile while delaying tumour growth and controlling blood pressure. Similarly, no efficacy or safety data are available for Stage D HFrEF.^15^ Without clearly demonstrated benefit of current or novel therapies for PGGL in Stage D HFrEF, aggressive management of HF and resection of PGGL prior to progression of HF are preferred when possible.

## Conclusion

We present a case of a patient with Stage D HF on inotropes due to mixed ischaemic heart disease and NICM in the setting of a PGGL who presents in cardiogenic shock. We describe medical management strategies for a patient with contraindications to surgical resection.

## Data Availability

No data were generated or analysed for this manuscript.
